# A Thermodynamic Approach to the Metaboloepigenetics of Cancer

**DOI:** 10.3390/ijms24043337

**Published:** 2023-02-07

**Authors:** Umberto Lucia, Thomas S. Deisboeck, Antonio Ponzetto, Giulia Grisolia

**Affiliations:** 1 Dipartimento Energia “Galileo Ferraris”, Politecnico di Torino, Corso Duca degli Abruzzi 24, 10129 Torino, Italy; 2 Department of Radiology, Harvard-MIT Martinos Center for Biomedical Imaging, Massachusetts General Hospital and Harvard Medical School, Charlestown, Boston, MA 02129, USA; 3 Department of Medical Sciences, University of Torino, Corso A.M. Dogliotti 14, 10126 Torino, Italy; 4 Dipartimento Scienza Applicata e Tecnologia, Politecnico di Torino, Corso Duca degli Abruzzi 24, 10129 Torino, Italy

**Keywords:** cancer, thermodynamics, metaboloepigenetics, epigenetics, transport phenomena

## Abstract

We present a novel thermodynamic approach to the epigenomics of cancer metabolism. Here, any change in a cancer cell’s membrane electric potential is completely irreversible, and as such, cells must consume metabolites to reverse the potential whenever required to maintain cell activity, a process driven by ion fluxes. Moreover, the link between cell proliferation and the membrane’s electric potential is for the first time analytically proven using a thermodynamic approach, highlighting how its control is related to inflow and outflow of ions; consequently, a close interaction between environment and cell activity emerges. Lastly, we illustrate the concept by evaluating the Fe${}^{2+}$-flux in the presence of carcinogenesis-promoting mutations of the TET1/2/3 gene family.

## 1. Introduction

The main aim of cancer appears to be continuous cell replication as well as spatial expansion of the tumour system itself, a process involving local invasion and distant metastasis [1]. As the scientific knowledge of complex systems increases, cancer emerges as a disease of the breakdown of the natural biological order within the body, a consequence of the malfunctioning of the controls of cell replication. In this context, an epigenetic approach could be useful in aiding our understanding of the processes involved. ‘Epigenetics’ is the biological science that studies phenotypic changes that do not involve alterations in the DNA sequence. Over the last few decades, evidence supporting the importance of gene-environment interactions for the regulation of gene expression and phenotypic outcome has mounted [2]. Furthermore, metabolism has been shown to affect gene expression: a review of the interaction between some metabolites and gene expression is summarised in Ref. [2]. Consequently, the link between energy metabolism and epigenetic control of gene expression can be analysed from the perspective of ‘metaboloepigenetics’.

We note that in 1956, cancer cells were first shown to be electrically different from normal cells [3]. Then, in 1969, in relation to the cell cycle phases, Cone Jr. pointed out that hyperpolarization represents a characteristic of the start of the M phase, introducing the hypothesis of a possible link between the membrane’s electric potential and cell cycle progression [4]. In addition, in 1970, he showed that membrane hyperpolarization was able to reversibly block the synthesis of DNA and mitosis [5], and in 1971, he reported that an increase in cancer cell proliferation could be ascribed to a lower than normal membrane potential [6]. These results have repeatedly been experimentally confirmed [7,8,9,10].

The fundamental role of the cellular membrane potential and its link to metaboloepigenetics represents an interesting topic of investigation. This link can be shown through the ion fluxes across the membrane, and its effect on gene activity can be studied in relation to the use of energy carried by the ions themselves. Indeed, living cell systems can use and transform energy in different forms:

- Mechanical energy, related to cellular movement, reorganization of intracellular structures, and changes of cell shape;
- Electrical energy, related to electron flow due to differences in voltage;
- Electromagnetic energy, related to thermal radiation, etc.;
- Chemical energy, related to biochemical reactions, but also to growth as an increase of molecules and biological structures;
- Heat transfer, as outflow due to wasted energy released into the cell microenvironment;
- Quantum energy, related to the structure of molecules and their quantum-level interactions.

Cell metabolism consists of thousands of chemical reactions which occur in organized sequences or metabolic pathways, such that they can lead from a high to low oxidative state in anabolism or vice versa in catabolism [11]. Thus, life is a complex biological phenomenon represented by numerous chemical, physical and biological processes, with the same physical laws governing processes in both animate and inanimate matter [12].

Thermodynamics is the discipline of physical science that allows us to study and interpret the evolution of any system in relation to energy use and conversion. As such, the aim of this paper is to develop an analysis of the metaboloepigenetic relationship from a thermodynamic viewpoint, in an effort to obtain a useful tool for cancer researchers and physicians to support the interpretation of experimental results in oncology.

## 2. Results

The fundamental results of this paper are expressed in Equation (12) and can be summarised as follows:

- Any change in the cell membrane’s electric potential generates entropy (σ); as such, this process is irreversible unless the cell consumes metabolites (to reverse the potential if needed). This proves the strict correlation between energy management and cell activity;
- Cell proliferation (dV/dt) is related to the membrane’s electric potential, confirming Cone Jr.’s experimental results [4,5,6];
- The change of the membrane’s electric potential can be controlled by the inflow and outflow of ions ($\sum_{i}\mu_{i}J$), which highlights the close interaction between environment and cell activity, as asserted by epigenetics;
- Any change in the cell membrane potential is related to energy management and ion concentration, as represented by the Nernst equation.

Applying these thermodynamics results to cancer, we note the fundamental role played by DNA (Deoxyribonucleic acid) methylation. As an example, we discuss mutations of the TET1/2/3 gene family which have been well characterized in driving hematological carcinogenesis [13]; recently, deregulated TET1/2/3 functions via multimodal mechanisms have also been shown in solid tumors. Indeed, disrupted TET1/2/3 catalytic activity determines the reconstitution of the methylation landscape, which plays a fundamental role in carcinogenesis. Ten-eleven translocation (TET) enzymes are a family of dioxygenases that iteratively catalyse 5-methylcytosine (5mC) oxidation and promote cytosine demethylation, thereby creating a dynamic methylation landscape [2], with the promotion of a cancer phenotype. The TET enzymes use α-ketoglutarate (α-KG) as cosubstrates to bind (Fe${}^{2+}$) in order to activate molecular oxygen; in particular, Fe${}^{2+}$ may work as a catalyst for the generation of ROS (reactive oxygen species) in pathological conditions such as carcinogenesis, inflammation, radiation and reperfusion injury. Indeed, Fe${}^{2+}$ overload has been associated with carcinogenesis (with major target genes, p16(INK4A) and p15(INK4B) tumor suppressor genes, which encode cyclin-dependent kinase inhibitors) in a ferric nitrilotriacetate-induced rat renal carcinogenesis model, in which the Fenton reaction was induced in the renal proximal tubules [14].

Thus, Fe${}^{2+}$ plays a fundamental role and our thermodynamic approach allows us to evaluate the iron fluxes through the cell membrane. To do so, we consider that the heat transfer from the cell to its environment occurs in a convective way, and as a consequence of (6), it follows that:

(1) $$\nabla \cdot J_{Q}=\alpha \frac{dA}{dV}\left(T-T_{0}\right)$$

where $\alpha \approx 0.023Re^{0.8}Pr^{0.35}\lambda /\langle R\rangle$ is the coefficient of convection, with λ≈0.6 W m${}^{-1}$ K${}^{-1}$ conductivity, Re≈0.2 the Reynolds number and Pr≈0.7 being the Prandtl number [15], *A* stands for the area of the cell membrane, *V* represents the cell volume.

Considering Equation (7), we can introduce the following simplifications:

- We consider the ideal case (σ=0 W m${}^{-3}$ K${}^{-1}$): it allows us to evaluate the maximum value of the ion fluxes;
- We consider the stationary state (ds/dt=0).

Consequently, it follows that:

(2) $$\begin{array}{cc} J_{{\mathrm{Fe}}^{+}} & =\frac{\ell \cdot \alpha }{\mu_{{\mathrm{Fe}}^{+}}\cdot \langle R\rangle }\left(T-T_{0}\right)=\\ & =\frac{0.004\times 10^{-6}\times 0.023\times 0.2^{0.8}\times 0.7^{0.35}\times 0.6}{1556.5\times 10^{3}\cdot {\langle R\rangle }^{2}}\left(T-T_{0}\right)=\\ & =\frac{3.45\times 10^{-18}[\mathrm{mol}s^{-1}]}{{\langle R\rangle }^{2}\left[m^{-2}\right]}=\frac{1.93\times 10^{-19}[\mathrm{kg}s^{-1}]}{{\langle R\rangle }^{2}\left[m^{-2}\right]} \end{array}$$

where ℓ≈0.004 μm is the depth of the cell membrane [16] and $\langle R\rangle$ denotes the mean radius of the cell, considered, in the first approximation, as a sphere, $\mu_{{\mathrm{Fe}}^{2+}}=1556.5$ kJ mol${}^{-1}$, and $T-T_{0}\approx 0.4$ °C [17]. The numerical result depends on the mean size of the cell. It follows that the (max) Fe${}^{2+}$-flux is of the order of $3.45\times 10^{-18}$ mol s${}^{-1}$m${}^{-2}$ = $1.93\times 10^{-19}$ kg s${}^{-1}$m${}^{-2}$, beyond which the cell would suffer damage.

This value refers to a single cell. To understand whether it is meaningful and in agreement with experimental results, we consider that in a human body there are approximately $30\times 10^{18}$ cells and also a comparable number of bacteria [16], with a radius (of mean value) of about $10^{-5}$ m. Consequently, considering a cell as a sphere, the total amount of iron mass can be evaluated per day (86,400 s) and yields a result of 20.6 mg${}_{{\mathrm{Fe}}^{2+}}$ kg${}_{hb}^{-1}$, where hb means ‘for an entire human body’. This numerical result must be compared with well-known results from etiology: ingestion of less than 20 mg kg${}^{-1}$ of elemental iron is nontoxic, while ingestion of 20 mg kg${}^{-1}$ to 60 mg kg${}^{-1}$ results in moderate symptoms, and ingestion of more than 60 mg kg${}^{-1}$ can lead to significant toxicity with severe morbidity and mortality [18,19].

## 3. Discussion

In 1942, Conrad Waddington introduced the concept of epigenetics to describe the developmental processes between genotype and phenotype [20]. As for cancer, the packaging of the genome and its regulation were thought to be the fundamental processes involved in the preservation of cellular health [21]. We add here a novel thermodynamic viewpoint to cancer metaboloepigenetics. Our results confirm the close interaction between cell activity and cellular microenvironment, and we analytically prove the link between cell proliferation and the membrane’s electric potential, controlled by the inflow and outflow of ions.

TET genes are frequently mutated in various cancers, and they play an important role in tumorigenesis due to their role in regulating DNA methylation and transcription [22]; indeed, cancer initiation and progression are related to modifications of DNA methylation [23]. TET1,2,3 oxidize the 5-methyl group of 5-methylcytosine (5mC) in DNA by involving oxygen and 2-oxoglutarate as substrates, and Fe${}^{2+}$ as a cofactor to yield 5-hydroxymethylcytosine (5-hmC), CO${}_{2}$, and succinate [24,25]. In our paper, the role of fluxes has been highlighted, and specifically, Fe${}^{2+}$ was evaluated using a nonequilibrium thermodynamic approach. Indeed, Fe${}^{2+}$ is a pro-oxidant agent that can produce ROS by reacting with hydrogen peroxide (H${}_{2}$O${}_{2}$). If saturation of the antioxidant system occurs then an excess of ROS determines lipid peroxidation, amino acid oxidation, loss of protein structure, and DNA damage, with related tissue damages. Moreover, lipid peroxidation can change the plasma membrane’s composition, fluidity and permeability, modifying the activity of integral proteins, particularly Na${}^{+}$,K${}^{+}$-ATPase and the Ca${}^{2+}$-ATPase [26], with an increase in Ca${}^{2+}$ intracellular concentration. In this context, the H${}^{+}$-ATPase plays a fundamental role, due to its function of inflowing positive charges into the cell. Recently, in relation to iron metabolism within the tumor microenvironment, the iron promotion of the production of reactive oxygen species was emphasized. This highlights the function of triggering ferroptosis (iron-dependent cell death) or supporting malignant transformation [27].

Our result, as obtained in Equation (12), represents the quantitative evaluation of the Fe fluxes required by cells to maintain the best thermodynamic conditions for supporting cellular viability. Theoretically, this value informs physicians to supplement current anticancer therapies with a defined amount of iron which sustains TET activities, in an effort to counter the impact of tumorigenesis-promoting TET mutations [28].

We note that, while we have focused our analysis on Fe${}^{2+}$ fluxes to showcase the utility of thermodynamic concepts in elucidating metaboloepigenetics, in reality, several other elements cross the cell membrane and should be of critical importance in maintaining homeostasis, each one in theory necessitating its own, specific Equation (2) description. However, while a combined analysis is beyond the scope of this paper, the fact that our numerical evaluations of Fe only come within ∼4.5% error when compared with experimental data (that implicitly include these other elements) gives credence to our stepwise approach.

In summary, as exemplified by iron and the TET loop, our approach presented here can aid in understanding the global patterns of epigenetic modifications in cancer.

## 4. Materials and Methods

As a consequence of the cancer system’s properties of heterogeneous clonal expansion, replicative immortality, patterns of longevity, rewired metabolic pathways, altered reactive oxygen species, evasion of death signals, and metastatic invasion [1,29,30], cancer can be modelled as an adaptive system based on natural selection that renders any single cancer cell independent of its neighbours [1]. Therefore, a thermodynamic approach emerges for modelling cells as open systems with the ability to convert metabolic energy into mechanical and chemical useful works, while heat discharges into their microenvironments. From a thermodynamic standpoint, metabolic energy represents the energetic inflow of a thermodynamic ‘cellular’ engine, with the following differences for the case of normal versus cancer cells, respectively [31]:

- For normal cells: the Krebs cycle, which consists of chemical reactions to convert stored energy through the oxidation of acetyl-CoA, using carbohydrates, lipids, and proteins;
- For cancer cells: the Warburg cycle, which consists of chemical reactions for specialised fermentation over the aerobic respiration pathway [32,33,34,35].

As referenced above, in 1971, Con Jr. showed that an increase in proliferation of cancer cells is caused by their lowered membrane potential, compared with the reference potential of non-cancerous, normal cells [6]. Consequently, the cytoplasmatic pH and the extracellular environment present a link to the cells’ membrane potential [36]. The electric potential difference, Δϕ, between the cytoplasm and the extracellular environment is evaluated with respect to the environment [37] by using the Goldman–Hodgkin–Katz equation [38,39,40]:

(3) $$\Delta \phi =\frac{RT}{F}\ln\left(\frac{P_{{\mathrm{Na}}^{+}}{\left[{\mathrm{Na}}^{+}\right]}_{\mathrm{outside}}+P_{K^{+}}{\left[K^{+}\right]}_{\mathrm{outside}}+P_{{\mathrm{Cl}}^{-}}{\left[{\mathrm{Cl}}^{-}\right]}_{\mathrm{outside}}}{P_{{\mathrm{Na}}^{+}}{\left[{\mathrm{Na}}^{+}\right]}_{\mathrm{inside}}+P_{K^{+}}{\left[K^{+}\right]}_{\mathrm{inside}}+P_{{\mathrm{Cl}}^{-}}{\left[{\mathrm{Cl}}^{-}\right]}_{\mathrm{inside}}}\right)$$

where [A] represents the concentration of the ion A, R=8.314 J mol${}^{-1}$K${}^{-1}$ is the universal constant of ideal gasses, *T* depicts the absolute temperature, F=96,485 C mol${}^{-1}$ is the Faraday constant, and *P* is the relative permeability [41,42,43], such that $P_{{\mathrm{Na}}^{+}}=0.04$, $P_{K^{+}}=1$ and $P_{{\mathrm{Cl}}^{-}}=0.45$ [41,42,43].

Cells can alter their membranes’ electric potential by changing the concentration of the different ions, i.e., by modulating ion inflow and outflow [44,45]; with particular regard to cancer cells, an increase in the Na${}^{+}$ intracellular concentration, with a K${}^{+}$ constant intracellular concentration [46], has been shown to lead to depolarization during malignant transformation of cells [7,47,48]. This experimental evidence demonstrates the fundamental role of the cell membrane’s electric potential for the control of malignant behaviour such as cell de-differentiation, proliferation, and migration [49,50,51].

Here, we propose a thermodynamic analysis of these processes to highlight the epigenetic and metaboloepigenetic conditions to support the treatment of cancer by conditioning ion fluxes. To do so, the Onsager phenomenological equations [52,53,54,55,56] must be considered:

(4) $$\left\{\begin{array}{c} J_{e}=-L_{11}\frac{\nabla \phi }{T}-L_{12}\frac{\nabla T}{T^{2}}\\ J_{Q}=-L_{21}\frac{\nabla \phi }{T}-L_{22}\frac{\nabla T}{T^{2}} \end{array}\right.$$

where $J_{e}$ depicts the current density [A m${}^{-2}$], $J_{Q}$ stands for the heat flux [W m${}^{-2}$], *T* is the living cell temperature, and $L_{ij}$ are the phenomenological coefficients, such that $L_{12}=L_{21}$ in the absence of magnetic fields, and $L_{11}\geq 0$ and $L_{22}\geq 0$, and $L_{11}L_{22}-L_{12}^{2}>0$ [52,53,54,55,56,57,58]: $L_{11}$ and $L_{22}$ represent the heat conductivity and the electrical conductivity, respectively, while $L_{12}$ and $L_{21}$ are cross coefficients, independent of both $L_{11}$ and $L_{22}$ [57,58].

Now, we consider that:

- The fluxes of an ion cause a variation in the concentration of the ion itself, balanced by the following equation [52,53]
  (5) $$\frac{dc_{i}}{dt}=-\nabla \cdot J_{i}$$
  where $c_{i}$ denotes the concentration of the *i*-th ion (Na${}^{+}$, K${}^{+}$, Ca${}^{2+}$, Cl${}^{-}$, etc.), *t* is the time, and $J_{i}$ stands for the current density of the *i*-th ion;
- The heat flux can be evaluated by the First Law of Thermodynamics as follows [52,53]:
  (6) $$\frac{du}{dt}=-\nabla \cdot J_{Q}$$
  where *u* represents the specific internal energy;
- The Second Law of Thermodynamics results in [59,60]
  (7) $$T\frac{ds}{dt}=-\nabla \cdot \left(J_{Q}-\sum_{i=1}^{N}\mu_{i}J_{i}\right)-\sum_{i=1}^{N}J_{i}\cdot \nabla \mu_{i}$$
  where *s* represents the specific entropy, *T* is the temperature, $J_{S}=J_{Q}-\sum_{i=1}^{N}\mu_{i}J_{i}$ denotes the contribution of the inflows and outflows, and $T\sigma =-\sum_{i=1}^{N}J_{i}\cdot \nabla \mu_{i}$ is the dissipation function [52], with μ being the chemical potential, defined as:
  (8) $$\mu_{i}={\left(\frac{\partial G}{\partial n_{i}}\right)}_{T,p,n_{k\neq i}}\approx \frac{G}{n_{i}}=g$$
  where *G* is the Gibbs energy, *g* represents the Gibbs molar specific energy, *n* is the number of moles, and *p* stands for the pressure. The entropy outflow σ is fundamental to generate order from disorder, as Schrödinger himself pointed out [61].

From Equation (5), we can state that an ion flux implies a variation in ion concentration on both sides of the membrane, with a related variation of the pH. Now, considering the Nernst equation [40]:

(9) $$Fd\phi =dg+2.3R\;T_{0}\;d\mathrm{pH}$$

where F=96,485 A s mol${}^{-1}$ is the Faraday constant and R=8.314 J mol${}^{-1}$ K${}^{-1}$ is the universal constant of ideal gas, it follows that a cell can control its membrane electric potential by managing ion flow through the membrane itself; but the change in the electric membrane potential is also related to the Gibbs free energy, which itself is related to the internal energy and entropy by its definition:

(10) G=U+pV−TS

Then, from Equation (6), it follows that [62]

(11) $$\int_{V}\frac{du}{dt}\;dV=\int_{V}\rho \;c\;\frac{dT}{dt}dV=-\int_{V}\nabla \cdot J_{Q}dV=-\dot{Q}$$

where $\rho \approx 10^{3}$ kg m${}^{-3}$ is the cell density, c≈4186 J kg${}^{-1}$ K${}^{-1}$ is the specific heat of the cell.

Finally, considering these results together with Equation (7), it follows that:

(12) $$F\frac{d\phi }{dt}=\frac{p}{n}\frac{dV}{dt}-\nabla \cdot \left(\sum_{i}\mu_{i}J_{i}\right)+T\sigma$$

which means that a change in the cell’s electric potential determines:

- The volume of the cell (dV/dt), which is related to cell proliferation;
- The fluxes of ions ($\mu_{i}J_{i}$), which are related to the metabolic requirements, respiration, communication, molecule formation and epigenetic effects;
- The heat discharged towards the environment (Tσ), due to irreversibility.

## Data Availability

The data used have been here evaluated and the reference to literature is quoted in the References.
